# Temperature-controlled surface adhesion in graphene materials: experimental trends, surfaces, and interfaces physical chemistry

**DOI:** 10.1039/d5ra03892h

**Published:** 2025-08-06

**Authors:** Tayssir Hamieh

**Affiliations:** a Faculty of Science and Engineering, Maastricht University P. O. Box 616 6200 MD Maastricht The Netherlands t.hamieh@maastrichtuniversity.nl; b Institut de Science des Matériaux de Mulhouse, Université de Haute-Alsace, CNRS, IS2M UMR 7361 F-68100 Mulhouse France; c Laboratory of Materials, Catalysis, Environment and Analytical Methods (MCEMA), Faculty of Sciences, Lebanese University P. O. Box 6573/14 Beirut Lebanon

## Abstract

Understanding the role of temperature in modulating surface adhesion properties of graphene and its derivatives is essential for their effective integration in nano- and optoelectronic devices. In this study, the temperature-dependent dispersive and polar components of work of adhesion was systematically investigated across graphene (G), reduced graphene oxide (rGO), and graphene oxide (GO), using inverse gas chromatography (IGC) and selected polar/nonpolar solvent interactions. Our results reveal a consistent hierarchy in adhesion energies (G > rGO > GO) and show that elevated temperatures significantly influence interfacial interactions by modifying surface energy components. Furthermore, the solvent-specific trends suggest a strong interplay between molecular polarity and surface functionalization. This study not only provides thermodynamic insights into graphene-based adhesion but also contributes to rational interface engineering in 2D materials under thermal fluctuation.

## Abstract 2

Temperature-tunable adhesion of graphene, GO, and rGO is explored across solid–vapor and solid–liquid interfaces. The findings proved controllable surface energy and interfacial mechanics, enabling future applications in fluidic devices and coatings.

## Graphical abstract

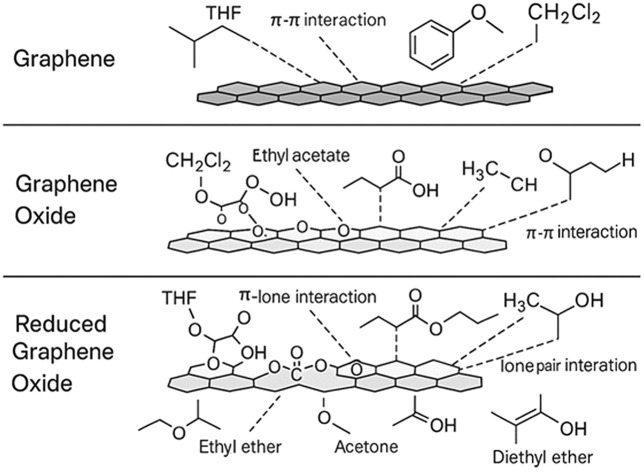

## Introduction

The London dispersive and polar thermodynamic surface properties of solid materials correlated to the intermolecular interactions between materials and adsorbents are of crucial interest in many scientific domains such as catalysis, adhesion, adsorption, coatings, friction, conduction, chemical engineering, and electronics. The temperature influences the surface properties of materials and their behavior when interacting with other materials or organic solvents. Indeed, the temperature affects the interactions between particles or molecules, and consequently, the adhesive, two-dimensional state, and surface properties of materials such as graphene, graphite, and carbon materials in several industrial applications. The thermodynamic surface properties of graphene are of great importance particularly in nanocomposites, nano-coating, and electrical nanodevices.^1^ The thermal and electrical properties of graphene were widely studied in the literature.^2–12^ Amanda *et al.*^13^ studied the effect of temperature on the structure and morphology of graphene. Whereas, Xiong *et al.*^14^ synthesized the graphene oxide (GO) and its reduction in electrochemically derived GO. The graphene and its derivatives were advantageously used for improving of the properties of cement-based building materials, including maneuverability, durability, and mechanical properties.^15–18^ Graphene can effectively improve the mechanical and electrical properties of cement-based materials due to its excellent tensile strength, thermal conductivity and electrical conductivity.^19^ Li *et al.*^19^ proposed the use of graphene in improving interfacial adhesion, electrical and thermal conductivity of concrete, absorbing heavy metal ions, and harvesting building energy. Graphene, as a typical two-dimensional nanometer material, has shown its unique application potential in electrical characteristics, thermal properties, and thermoelectric properties by virtue of its novel electronic structure.^20,21^ Furthermore, the interface between two-dimensional (2D) materials such as graphene and its chemically modified forms—graphene oxide (GO) and reduced graphene oxide (rGO) is the cornerstone of modern nanotechnology.^22^

However, despite their intrinsic chemical versatility, the temperature-dependent nature of their surface adhesion work remains insufficiently explored. Indeed, the work of adhesion (*W*_a_), an important thermodynamic variable governing the interfacial interactions of solvents on solid surfaces, quantifies the energy required to separate two phases in contact.^23,24^ The molecular mechanisms and thermodynamics for determining solvent adhesion to solid material surfaces are essential for controlling wettability, solvent adsorption, and interfacial interactions.^25,26^ The concept of thermodynamic “work of adhesion”, *W*_a_, was first introduced by Harkins^27^ who used the various surface tensions of liquids adhering to a solid. The combination of Harkins's concept,^27^ Young–Dupré equation,^28,29^ Fowkes,^30^ Owens–Wendt,^31^ and van Oss *et al.*,^32,33^ led to the determination of the work of adhesion taking in consideration the dispersive, polar, and acid–base interactions.^30–33^ Several experimental techniques, such as dynamic contact angle technique, isothermal titration calorimetry (ITC), and inverse gas chromatography (IGC), were used for evaluating the adhesion works and contributing to the quantification of intermolecular interactions.^24,34^

Adhesion plays a critical role in applications such as flexible electronics, membrane-based sensing, nanofluidics, and heterogeneous integration. Graphene's adhesion to various substrates has been attributed primarily to van der Waals forces, whereas GO and rGO exhibit complex interactions involving hydrogen bonding and dipolar forces due to surface oxygen functionalities.^35^ The interplay of these forces evolves with temperature, affecting not only contact energy but also interfacial mobility, roughness adaptation, and phonon coupling.^36^

Recent studies demonstrate that the adhesion of 2D materials can be “gas-like,” characterized by a temperature-sensitive entropic contribution that decreases linearly with increased thermal vibration.^37^ Moreover, functional group density and orientation on GO/rGO surfaces are thermally active and can reconfigure interfacial potential landscapes.^38^

Temperature-controlled surface energy variation is not merely of academic interest—it directly affects material transfer yield, contact resistance, and durability in heterostructures.^39^ While contact mechanics and adhesion of pristine graphene have been modeled thermodynamically, empirical and solvent-specific trends for functionalized graphene variants are scarce.^40^

Some surface properties of graphene oxide (GO) and graphene (rGO) were determined by Dai *et al.*^41^ using the inverse gas chromatography (IGC) technique at infinite dilution. However, these results cannot be considered as accurate due to the wrong hypothesis admitted by Dai *et al.*^41^ supposing the surface area and the London dispersive surface energy of *n*-alkanes as constant independent from the temperature. Indeed, our previous works^42–46^ proved a strong effect of the temperature on the surface area of organic molecules and gave the variations of the surface area and the London dispersive surface energy of the different solvents as a function of temperature. The same previous errors were committed by Lee *et al.*^47^ when determining the London dispersive and polar surface of graphene materials using the classic chromatographic methods by neglecting the temperature effect on the surface area and surface tension of solvents. The IGC technique at infinite dilution was used during the last fifty years for the determination of surface properties of solid materials such as oxides, polymers, metals, or fibers.^48–72^

In a previous study,^73^ the London dispersive and polar surface properties of different graphene and carbon materials were determined as a function of temperature by using our new chromatographic approach based on the Hamieh thermal model.^42–46^ On the other hand, some specific surface chemistries and morphologies of graphenes that directly influence their interfacial adhesion properties were studied in the literature. Ferrari *et al.*^74^ and Bunch *et al.*^75^ have confirmed the monolayer and monocrystalline structure of graphene (G) with minimal defects and high in-plane ordering. Whereas Dreyer *et al.*^76^ and Lerf *et al.*^77^ revealed an oxygen-to-carbon (O/C) atomic ratio of 0.45, indicating a moderate-to-high oxidation level and showing the presence of hydroxyl, epoxy, and carboxyl functional groups. The reduced graphene oxide (rGO) was investigated by Eda *et al.*^78^ and Stankovich *et al.*,^79^ showing that the O/C ratio decreased to approximately 0.12, indicating partial removal of oxygenated groups and a sharp reduction in OH and COOH features, though some residual carbonyl groups remained.

The temperature-dependent surface adhesion behavior of graphene materials plays a pivotal role in the design and optimization of microfluidic and nanofluidic systems, where precise control of fluid–surface interactions is critical. In particular, tunable adhesion at the solid–liquid or solid–vapor interface influences droplet mobility, flow resistance, and interfacial slip, enabling applications in thermal regulation, self-cleaning surfaces, and active fluid transport. Graphene coatings on substrates have been shown to enhance dropwise condensation and heat transfer by up to fourfold relative to conventional coatings, while offering ultrathin, thermally stable surfaces with minimal added resistance.^79^ Furthermore, bioinspired graphene–PDMS patterned surfaces have demonstrated reversible wettability switching between hydrophobic and hydrophilic states between 0 °C and 200 °C, enabling directed droplet transport and fog harvesting in engineered fluidic platforms.^80^ At the nanoscale, graphene's adhesion mechanics—dominated by van der Waals interactions and membrane flexibility—are critical to interfacial slip and droplet behavior in confined fluidic systems.^81^ Recent studies further show that graphene's wettability can be dynamically altered by external stimuli such as temperature or plasma exposure, resulting in reversible modulation of surface energy and adhesion properties.^82^ Finally, graphene-based open microfluidic channels engineered *via* laser patterning exploit these tunable surface forces to passively transport fluids without pumps, illustrating direct applications in lab-on-chip device technology.^83^

This study aims to perform a comprehensive, solvent-resolved thermodynamic analysis of adhesion behaviors of graphene, rGO, and GO by varying the temperature. New theoretical models that have been validated by several recent works based on the Hamieh thermal model^41–45^ and the new separation method using the London dispersive and polar free energy of adsorption^70,71^ were applied. By combining the new chromatographic models with quantitative work of adhesion calculations, a clearer picture of how thermal effects alter interfacial physics in 2D materials is given.

## Materials and methods

### Materials and solvents

Different organic solvents were used as probes to determine the London dispersive and polar surface properties of the different solid materials. The *n*-alkanes such as *n*-hexane, *n*-heptane, *n*-octane, and *n*-nonane were chosen as non-polar solvents. Whereas the polar organic molecules used were the Lewis acid molecules such as carbon tetrachloride (CCl_4_), chloroform (CHCl_3_), and dichloromethane (CH_2_Cl_2_); the amphoteric solvents such as acetone and acetonitrile, and the Lewis basic molecules such as ethyl acetate, diethyl ether, and tetrahydrofuran (THF). All solvents and graphene (G), graphene oxide (OG), and reduced graphene (rOG) materials were purchased from Fisher Scientific (Beirut, Lebanon).

### Chromatographic measurements

Experimental measurements were carried out on a commercial Focus GC gas chromatograph equipped with a flame ionization detector (Sigma-Aldrich, St. Quentin Fallavier, France). The graphene particles were poured into a stainless-steel column with a 2 mm inner diameter and a length of 20 cm. The column was packed with 1 g of solid materials under temperatures varying from 313.15 K to 373.15 K, and 473.15 K for the injector and detector. The retention time *t*_R_ was measured with a standard deviation lower than 1% in all experiments. The infinite dilution of the injected solvents was realized by using 1 μL Hamilton syringes and injecting extremely diluted quantities of the vapor probe.^71,73^ The columns containing the graphene particles were preconditioned at 130 °C overnight to ensure the total desorption of water molecules or any other residual impurities. The injection of the different probes into the column experimentally led to the values of the retention time *t*_R_ of the adsorbed solvents and the dead reference retention time *t*_0_ of a non-adsorbing probe such as methane, necessary for the determination of the net retention volume *V*_n_ of the probes using eqn (1):

1 *V*_n_ = *jD*_c_(*t*_R_ − *t*_0_)

where *D*_c_ is the corrected flow rate of the carrier gas (helium) and *j* is a correction factor which takes into account the compression of the gas. *D*_c_ and *j* are respectively given by relations (2) and (3):2
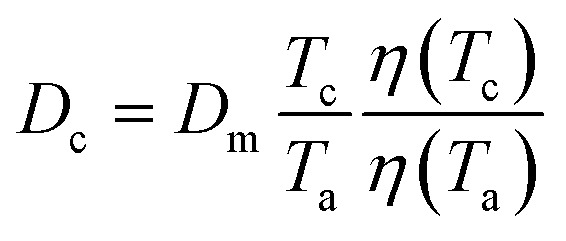
3
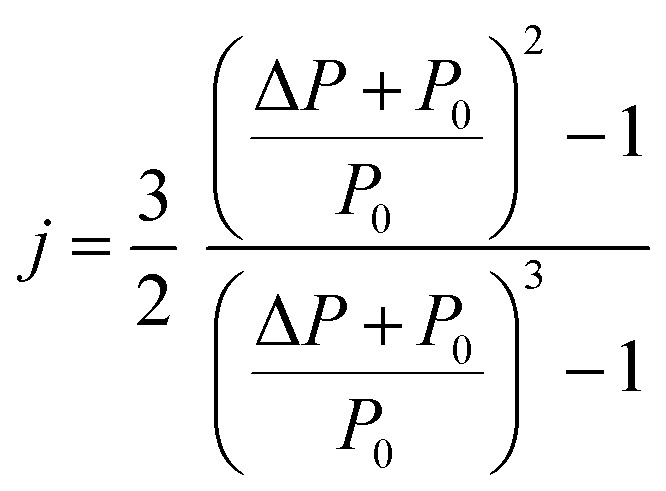
where *D*_m_ is the measured flow rate, *T*_c_ the column temperature, *T*_a_ the room temperature, *η*(*T*) the gas viscosity at temperature *T*, *P*_0_ the atmospheric pressure and Δ*P* the pressure variation.

The determination of the retention volume leads to the variations of the standard free energy Δ*G*^0^_a_ of adsorption of organic solvents on the different solid surfaces as a function of temperature using the fundamental equation of inverse gas chromatography:

4 −Δ*G*^0^_a_(*T*) = *RT* ln *V*_n_(*T*) + *C*(*T*)

where *R* is the perfect gas constant and *C*(*T*) a constant depending on temperature and interaction between the solvents and solid materials.

Δ*G*^0^_a_ is equal to the London dispersive free energy Δ*G*^d^_a_ and the polar interaction energy Δ*G*^p^_a_ of adsorption of solvents on solid surfaces:

5 Δ*G*^0^_a_ = Δ*G*^d^_a_ + Δ*G*^p^_a_

The two previous contributions of the free energy of adsorption were separately determined in previous works using the London dispersion interaction equation.

### Thermodynamic methods

#### Surface energy parameters of graphenes

The London dispersive surface energy *γ*^d^_s_(*T*) of the different graphenes was determined against the temperature by applying the Hamieh thermal model^42–46^ who proposed the expressions of the surface area *a*(*T*) of *n*-alkanes as a function of temperature. This new model criticized the classic models that supposed constant values of the surface area and the London dispersive surface energy of solvents adsorbed on solid materials and gave more accurate values of *γ*^d^_s_(*T*) of graphenes *versus* the temperature. Using Fowkes eqn (6):6
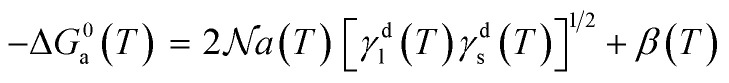
Where *γ*^d^_l_(*T*) is the London dispersive component of the surface energy of the solvent, 
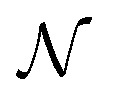
 the Avogadro number, and *β*(*T*) a constant depending on the temperature and the solid material.

In the case of non-polar solvents such as *n*-alkanes adsorbed on solid surfaces, eqn (6) can be written as follows:7
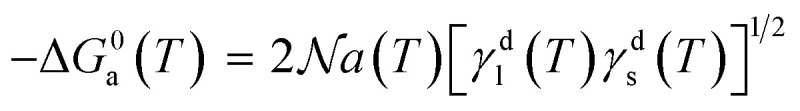


The variations of *a*(*T*) and *γ*^d^_l_(*T*) of *n*-alkanes adsorbed on graphenes as a function of temperature led to accurate values of the London dispersive surface energy of different graphenes. Whereas the dispersive free energy Δ*G*^0^_a_(*T*) and the polar free energy Δ*G*^p^_a_(*T*) of different organic solvents adsorbed on graphene, GO, and rGO, were separated using our new methodology based on the London interaction energy equation. By applying the Van Oss *et al.*‘s method,^33^ the Lewis acid *γ*_s_^+^, and base *γ*_s_^−^ surface energies of graphenes were obtained by choosing two polar solvents such as ethyl acetate and dichloromethane and using eqn (8) given −Δ*G*^p^_a_(*T*):8
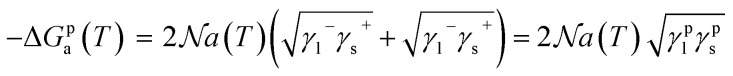
where *γ*_l_^+^ and *γ*_l_^−^ are respectively the Lewis acid and base surface energies of the polar solvent, and *γ*^p^_l_ and *γ*^p^_s_, respectively, the polar surface energy of the solvent and the graphene.

The polar surface energy of the solvent is obtained from eqn (8), while the polar surface energy *γ*^p^_s_(*T*) of graphenes was determined by the following equation:9
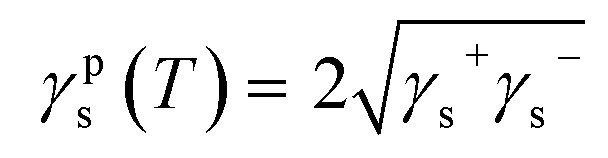
while the total surface energy *γ*_s_(*T*) of graphenes can be obtained from eqn (10):

10 *γ*_s_(*T*) = *γ*^d^_s_(*T*) + *γ*^p^_s_(*T*)

#### Relation between work of adhesion and surface energy

The work of adhesion *W*_a_ of a liquid of surface tension *γ*_l_ on a homogeneous, non-deformable, and isotropic solid surface can be defined by eqn (6):11
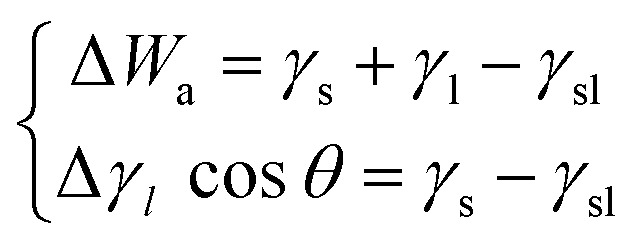
where *γ*_sl_ is the solid–liquid interface tension and *θ* the contact angle formed between the liquid drop and the plan solid surface which was first proposed by Young.^28^

However, Dupré^17^ using Young's equation^28^ gave the work of adhesion by eqn (12):

12 *W*_a_ = *γ*_l_(1 + cos* θ*)

While Fowkes^30^ gave a new expression relative to the dispersive work of adhesion *W*^d^_a_ by taking the geometric mean of the dispersive components of the solid and liquid:13
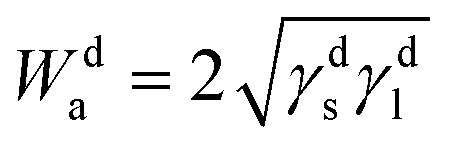


By considering the polar interaction, Fowkes^30^ gave the new expression of the work of adhesion:14
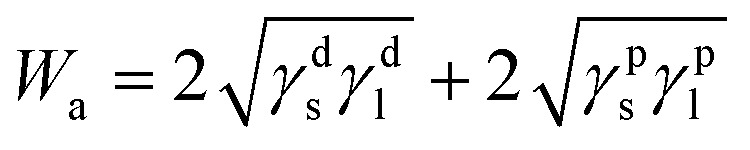


Finally, the work of adhesion can be written as follows:15
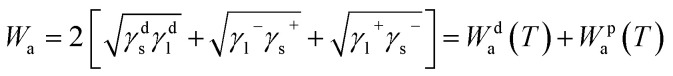


Knowing the different surface energy components of graphene materials previously determined,^73^ the dispersive *W*^d^_a_(*T*), polar *W*^p^_a_(*T*)and total *W*_a_(*T*) works of adhesion were determined using eqn (8) and (13)–(15).

## Results

### London Dispersive surface energy of graphenes

The London dispersive surface energy of graphene, graphene oxide, and reduced graphene oxide was determined using the Hamieh thermal model that gave the variations of the surface area and *γ*^d^_l_ of solvents as a function of temperature. It was previously proved that the methods of Schultz *et al.* and Dorris–Gray, supposing the surface area and *γ*^d^_l_ of solvent molecules as constant, are inaccurate and cannot be used to determine the London dispersive surface energy, nor to evaluate the polar interactions. Applying the Hamieh thermal model^42–46^ and the Fowkes equation,^30^ the *γ*^d^_s_(*T*) values of the different graphene materials against the temperature were determined by the slope of the straight line obtained by representing the variations in *RT* ln *V*_n_(*T*) of *n*-alkanes adsorbed on graphenes as a function of 
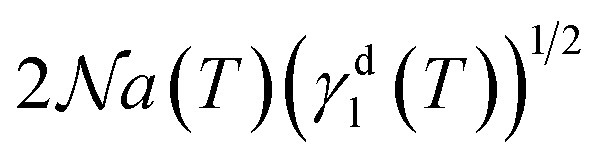
.^73^ The variations in *γ*^d^_s_(*T*) of graphenes were given in Table 1 at different temperatures.

**Table 1:** Values of the London dispersive surface energy *γ*^d^_s_ (in mJ m^−2^) of graphene, graphene oxide, and reduced graphene oxide at various temperatures

*T* (K)	Graphene	Graphene oxide	Reduced graphene oxide
313.15	279.19	118.24	150.97
323.15	261.11	109.29	147.26
333.15	243.30	100.60	143.33
343.15	225.78	92.15	139.24
353.15	208.54	83.95	134.93
363.15	191.63	76.03	130.33
373.15	175.03	68.36	125.48

Table 1 showed that *γ*^d^_s_ of graphenes linearly decreases as the temperature increased. The lowest *γ*^d^_s_ was obtained with the graphene oxide, whereas the highest value was shown with the graphene. It seems that the oxidation of graphene decreases the values of *γ*^d^_s_ to about 40% of its initial value, while the *γ*^d^_s_ variations of reduced graphene oxide decreased until 54% at 313.15 K to 71.7% at 373.15 K. The various graphenes can be classified in increasing order of the London dispersive surface energy as follows:Graphene oxide < Reduced graphene oxide < Graphene

The increasing order of London dispersive surface energy can be explained based on their chemical structure, electronic properties, and surface morphology, which directly affect the van der Waals (London dispersion) interactions. London dispersion forces are a type of van der Waals force, arising from instantaneous dipole-induced dipole interactions. These forces increase with surface electron density and polarizability. The dispersive component of surface energy is closely linked to π–π stacking ability, sp^2^ character, and electronic delocalization. The Graphene oxide (GO) structure is heavily oxidized with abundant oxygen-containing hydroxyl, epoxide, carbonyl, and carboxyl groups. This 2D material exhibits high disruption of sp^2^ domains and many sp^3^ hybridized carbons due to its functionalization with low surface π-electron delocalization. This gives weak London dispersion forces due to reduced polarizability and disrupted π-system leading to the lowest London dispersive surface energy. Whereas the reduced graphene oxide (rGO) is partially restored sp^2^ network, but with defects and residual oxygen groups with moderate π-delocalization and less polarity than GO. However, the graphene (G) structure presents 2D sheet of sp^2^-bonded carbon atoms with pure sp^2^ hybridization, highly delocalized π-electron cloud, non-polar hydrophobic material, and strongest London dispersion forces due to high polarizability and dense, continuous π-electron system, and consequently, the graphene exhibits the highest London dispersive surface energy *γ*^d^_s_.

### Polar surface energy of graphenes

The polar free energy of adsorption of the various solvents on graphene materials were determined as a function of temperature in a previous work.^73^ This led to the variations of the polar acid *γ*_s_+(*T*), base *γ*_s_−(*T*) surface energies, and the polar surface energy *γ*^p^_s_(*T*) of graphenes G, GO, and rGO *versus* the temperature. The results were presented in Table 2.

**Table 2:** Variations in base surface energy *γ*_s_−(*T*), acid surface energy *γ*_s_+(*T*), polar surface energy *γ*^p^_s_(*T*), total surface energy *γ*_s_(*T*) of graphenes (in mJ m^−2^) *versus* the temperature

Temperature *T* (K)	313.15	323.15	333.15	343.15	353.15	363.15	373.15
**Base surface energy *γ***_**s**_^−^ **(*T*) of graphenes (mJ m^−^** ^**2**^ **)**							
Graphene	73.55	68.61	63.95	59.53	55.36	51.42	47.70
Reduced graphene oxide	70.30	63.58	57.32	51.50	46.10	41.09	36.46
Graphene oxide	20.29	19.69	19.12	18.58	18.05	17.53	17.03

**Acid surface energy *γ***_**s**_^+^ **(*T*) of graphenes (mJ m^−^** ^**2**^ **)**							
Graphene	167.08	162.07	157.32	152.79	148.51	144.41	140.52
Reduced graphene oxide	103.34	98.87	94.69	90.70	86.96	83.44	80.07
Graphene oxide	5.32	7.25	9.41	11.79	14.29	16.93	19.68

**Polar surface energy *γ*** ^**p**^_**s**_ **(*T*) of graphenes (mJ m** ^**−2**^ **)**							
Graphene	221.71	210.91	200.61	190.75	181.34	172.35	163.73
Reduced graphene oxide	170.47	158.57	147.35	136.69	126.62	117.11	108.06
Graphene oxide	20.78	23.91	26.84	29.60	32.12	34.45	36.61

**Total surface energy *γ***_**s**_ **(*T*) of graphenes (mJ m^−^** ^**2**^ **)**							
Graphene	500.90	472.02	443.90	416.53	389.88	363.98	338.77
Reduced graphene oxide	321.4	305.8	290.7	275.9	261.6	247.4	233.5
Graphene oxide	139.0	133.2	127.4	121.7	116.1	110.5	105.0

Table 2 showed a highest acid–base surface energy character of graphene, followed by rGO, and GO. This is due to the polarizable electron cloud of graphene, even though it's chemically inert—it behaves like a strong polarizable surface, not because of functional groups, but due to delocalized π-electrons. Whereas rGO bridges the gap, exhibiting intermediate polarity and tunable surface chemistry depending on reduction extent. While the GO's polar behavior is governed by surface functionalities, which become more active with temperature, especially in acid–base interactions.

### Polar surface energy of organic solvents adsorbed on graphenes

The polar surface energy *γ*^p^_l_(*T*) of solvents adsorbed on the different graphene materials were obtained using eqn (3). The results given in Table S1 showed an important effect of temperature on *γ*^p^_l_(*T*) of solvents.

The various solvents adsorbed on graphene were classified in increasing order of polar surface energy as follows:Dichloromethane < THF < Ethyl acetate < Acetone < Diethyl ether < Acetonitrile

The above order proved that the solvents with increasing dipole moment and hydrogen-bonding capacity showed stronger polar interaction. The graphene interacts weakly *via* polar forces, so more strongly polar solvents (*e.g.*, acetonitrile) stand out in polar interactions.

In the case of reduced graphene oxide (rGO), the following increasing order of *γ*^p^_l_(*T*) was obtained:Dichloromethane < Acetonitrile < THF < Acetone < Ethyl acetate < Diethyl ether

It was observed that acetonitrile moves lower in polarity rank compared to graphene. Indeed, rGO has a complex surface with both polar and nonpolar domains. Solvents with dual character (*e.g.*, ethers) can form more stable interactions due to both dipolar alignment and weak H-bonding with residual oxygen groups. However, one obtained the following order of solvents adsorbed on graphene oxide (GO):Dichloromethane < THF < Diethyl ether < Acetone < Ethyl acetate < Acetonitrile

This order showed a strongest polar interaction with acetonitrile, a highly polar aprotic solvent. In fact, GO has abundant polar groups (–OH, –COOH, epoxides) that interact *via* dipole–dipole and hydrogen bonding, favoring polar solvents.

### Work of adhesion of solvents on graphenes

**Fig. 1:**
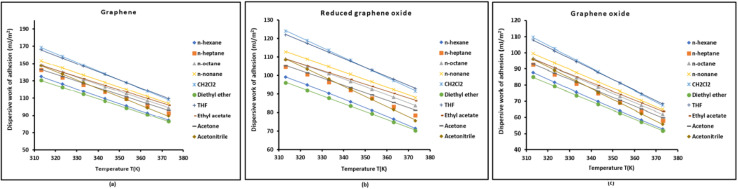
Variations of the dispersive work of adhesion *W*^d^_a_(*T*) of solvents on graphenes *versus* the temperature. Graphene (a), reduced graphene oxide (b), and graphene oxide (c).

The variations of the dispersive *W*^d^_a_(*T*), polar *W*^p^_a_(*T*), and total work *W*_a_(*T*) of adhesion of solvents on graphene surfaces as a function of temperature were obtained using eqn (8)–(10) and the different values of London dispersive and polar surface energies of solvents, and graphenes. The values of the dispersive work of adhesion *W*^d^_a_(*T*) of solvents on graphenes were showed in Table S2 at different temperatures. Whereas the corresponding curves of *W*^d^_a_(*T*) *versus* the temperature were plotted in Fig. 1.

Fig. 1 and Table S2 clearly showed the same following increasing order of the work of adhesion of solvents on the different graphenes:Diethyl ether < *n*-Hexane < *n*-Heptane < Acetone < Acetonitrile < Ethyl acetate < *n*-octane < *n*-nonane < THF < CH_2_Cl_2_

Indeed, diethyl ether is characterized by its low polarity and lowest dispersive surface energy, and then very poor adhesion for all graphene materials. Now, the *n*-alkanes from *n*-hexane to *n*-nonane are purely dispersive, increasing the dispersive work of adhesion *W*^d^_a_(*T*) with chain length due to greater van der Waals contact area. Acetone and acetonitrile are polar aprotic solvents with relatively low dispersive surface energy. Whereas, ethyl acetate has both dispersive and moderately polar surface energy, and provides better adhesion. The higher dispersive surface energy of tetrahydrofuran (THF) and better matching to carbon surfaces result in higher *W*^d^_a_. Though dichloromethane is moderately polar, it has a relatively high dispersive surface energy, explaining its high adhesion with graphene surfaces.

Even though values of *W*^d^_a_(*T*) differ for graphene surfaces with the highest value for graphene followed by reduced graphene oxide, and graphene oxide, the solvent ranking remains constant because:

1. The dispersive component *W*^d^_a_ of adhesion work which mainly depends on *γ*^d^_s_ of graphene which is constant for each graphene at a fixed temperature, and on *γ*^d^_l_ of solvent which varies by solvent. The solvent then dominates the trend.

2. The ranking reflects increasing *γ*^d^_l_ and/or increasing molecular size/interaction area of the solvents.

3. Polar solvents (*e.g.* acetone, acetonitrile) appear earlier in the ranking because their *γ*^d^_l_ is relatively low, despite having dipoles.

**Fig. 2:**
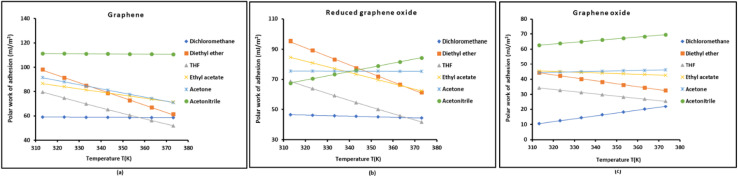
Variations of the polar work of adhesion *W*^p^_a_(*T*) of solvents on graphenes *versus* the temperature. Graphene (a), reduced graphene oxide (b), and graphene oxide (c).

The determination of polar work of adhesion *W*^p^_a_(*T*) of solvents on graphenes *versus* the temperature was given in Table S3. The variations of *W*^p^_a_(*T*) were drawn in Fig. 2. The results in Table S3 and Fig. 2 allowed giving the different solvents in increasing order of *W*^p^_a_(*T*) on graphenes:

For graphene:CH_2_Cl_2_ < THF < Ethyl acetate < Acetone < Diethyl ether < Acetonitrile

For reduced graphene oxide:CH_2_Cl_2_ < Acetonitrile < THF < Acetone < Ethyl acetate < Diethyl ether

For graphene oxide:CH_2_Cl_2_ < THF < Diethyl ether < Acetone < Ethyl acetate < Acetonitrile

The above rankings reflect how well the polarity of graphenes matches the polarity of the solvent. The graphene is considered as non-polar surface. This justifies the very low its polar surface energy *γ*^p^_s_. Even highly polar solvents (like acetonitrile) don't interact much the polar work of adhesion *W*^p^_a_(*T*) remains low, but small differences emerge from solvent polarity. Acetonitrile has the highest polar surface energy *γ*^p^_l_ and therefore the highest polar adhesion with G. Whereas the reduced graphene oxide (rGO) is characterized by intermediate *γ*^p^_s_ and partial removal of oxygen groups. The polar work of adhesion *W*^p^_a_(*T*) now depends more subtly on both *γ*^p^_s_ and *γ*^p^_l_. Even if acetonitrile is a polar solvent, however, it has low hydrogen-bonding ability leading to low polar adhesion with rGO. while Diethyl ether, though weakly polar, can interact through lone pairs and π-donation on oxygen and give relatively high polar work of adhesion *W*^p^_a_. The graphene oxide exhibiting the highest *γ*^p^_s_ due to hydroxyl, epoxide, carboxyl groups, interacts strongly with polar solvents, especially hydrogen-bond donors and acceptors. Thus, *W*^p^_a_ increases sharply with solvents like acetonitrile and ethyl acetate, while diethyl ether which is weakly polar (only lone pairs) leads to lower interaction than acetone.

In conclusion, the different molecular interactions (dipole–dipole, H-bonding, lone pair interaction) govern how polar work of adhesion varies across graphene surfaces. The trend inversion between rGO and GO shows the sensitivity of *W*^p^_a_ to subtle changes in surface chemistry. Finally, acetonitrile interacts strongly with GO but poorly with rGO, showing how hydrophilicity and donor–acceptor capacity influence polar interactions.

The total work of adhesion *W*_a_(*T*) of solvents on graphenes was obtained by summing the London dispersive and polar works of adhesion as a function of temperature. The linear variations of *W*_a_(*T*) were plotted *versus* the temperature in Fig. S1. The results clearly showed the highest values of the work of adhesion of polar solvents compared to those of *n*-alkanes due the highest polar interactions of polar molecules with graphenes. The linear relations of the work of adhesion *W*_a_(*T*) of different solvents on graphenes as a function of temperature were given in Table 3. It was deduced that *W*_a_(*T*) can be thermodynamically written as:

16 *W*_a_(*T*) = Δ*H*_S_ − *T*Δ*S*_S_

where Δ*S*_S_ and Δ*H*_S_ are respectively the surface entropy and the surface enthalpy of adhesion work of solvents on graphenes.

**Table 3:** Equations of total work of adhesion *W*_a_(*T*) (mJ m^−2^) of the various organic solvents on graphenes, with the linear regression coefficients, the surface entropy Δ*S*_S_ (mJ m^−2^ K^−1^), and the surface enthalpy Δ*H*_S_ (mJ m^−2^) of adhesion work, and the maximum temperature *T*_Max_

Solvents	*W*_a_(*T*)	Δ*S*_S_	Δ*H*_S_	*T*_Max._ (K)	*R* ^2^
**Graphene**					
*n*-Hexane	*W*_a_(*T*) = −0.843*T* + 398.73	0.843	398.73	473.05	0.9999
*n*-Heptane	*W*_a_(*T*) = −0.828*T* + 401.62	0.828	401.62	484.93	0.9999
*n*-Octane	*W*_a_(*T*) = −0.823*T* + 405.74	0.823	405.74	493.30	0.9999
*n*-Nonane	*W*_a_(*T*) = −0.822*T* + 410.53	0.822	410.53	499.49	1.0000
CH_2_Cl_2_	*W*_a_(*T*) = −1.016*T* + 545.64	1.016	545.64	536.84	0.9999
Diethyl ether	*W*_a_(*T*) = −1.407*T* + 668.69	1.407	668.69	475.23	0.9997
THF	*W*_a_(*T*) = −1.399*T* + 683.1	1.399	683.1	488.24	0.9998
Ethyl acetate	*W*_a_(*T*) = −1.017*T* + 552.89	1.017	552.89	543.49	1.0000
Acetone	*W*_a_(*T*) = −1.129*T* + 587.95	1.129	587.95	520.86	0.9999
Acetonitrile	*W*_a_(*T*) = −0.984*T* + 567.1	0.984	567.1	576.15	1.0000

**Reduced graphene oxide**					
*n*-Hexane	*W*_a_(*T*) = −0.463*T* + 244.46	0.463	244.46	528.11	0.9995
*n*-Heptane	*W*_a_(*T*) = −0.437*T* + 241.69	0.437	241.69	553.57	0.9996
*n*-Octane	*W*_a_(*T*) = −0.421*T* + 241.07	0.421	241.07	572.88	0.9996
*n*-Nonane	*W*_a_(*T*) = −0.411*T* + 241.68	0.411	241.68	587.89	0.9996
CH_2_Cl_2_	*W*_a_(*T*) = −0.578*T* + 351.51	0.578	351.51	608.68	0.9996
Diethyl ether	*W*_a_(*T*) = −0.999*T* + 504.22	0.999	504.22	504.57	0.9999
THF	*W*_a_(*T*) = −0.936*T* + 483.61	0.936	483.61	516.73	1.0000
Ethyl acetate	*W*_a_(*T*) = −0.743*T* + 426.15	0.743	426.15	573.71	1.0000
Acetone	*W*_a_(*T*) = −0.405*T* + 307.74	0.405	307.74	760.79	0.9996
Acetonitrile	*W*_a_(*T*) = −0.269*T* + 260.83	0.269	260.83	968.55	0.9954

**Graphene oxide**					
*n*-Hexane	*W*_a_(*T*) = −0.585*T* + 270.74	0.585	270.74	462.88	0.9998
*n*-Heptane	*W*_a_(*T*) = −0.579*T* + 273.76	0.579	273.76	472.90	0.9998
*n*-Octane	*W*_a_(*T*) = −0.578*T* + 277.28	0.578	277.28	479.81	0.9998
*n*-Nonane	*W*_a_(*T*) = −0.580*T* + 281.07	0.580	281.07	484.94	0.9998
CH_2_Cl_2_	*W*_a_(*T*) = −0.513*T* + 280.66	0.513	280.66	547.10	0.9998
Diethyl ether	*W*_a_(*T*) = −0.748*T* + 363.28	0.748	363.28	485.54	0.9997
THF	*W*_a_(*T*) = −0.803*T* + 393.34	0.803	393.34	489.90	0.9997
Ethyl acetate	*W*_a_(*T*) = −0.588*T* + 325.73	0.588	325.73	553.87	0.9999
Acetone	*W*_a_(*T*) = −0.526*T* + 302.00	0.526	302.00	574.47	0.9998
Acetonitrile	*W*_a_(*T*) = −0.558*T* + 333.07	0.558	333.07	597.33	0.9998

The deduced values of Δ*H*_S_ and Δ*S*_S_ of the different organic solvents on graphenes were given in Table 3, including the corresponding values of the maximum temperature *T*_Max_ defined as follows:

17 *T*_Max_ = Δ*H*_S_/Δ*S*_S_

Table 3 showed that the values of Δ*H*_S_ of the solvents adsorbed on graphenes linearly depends on the surface entropy of adhesion work Δ*S*_S_. Equations Δ*H*_S_ = *f*(Δ*S*_S_) of the different graphenes were given in Table 4. A new surface temperature *T*_S_ of material was defined.

**Table 4:** Equations of Δ*H*_S_ as a function of Δ*S*_S_ of the different modified copolymers with the values of *T*_S_ and the corresponding linear regression coefficients

2D materials	Equations Δ*H*_S_ = *f*(Δ*S*_S_)	*T*_S_
Graphene	Δ*H*_S_ = 471.03 Δ*S*_S_ + 38.517	471.03
Reduced graphene oxide	Δ*H*_S_ = 406.46 Δ*S*_S_ + 100.21	406.46
Graphene oxide	Δ*H*_S_ = 370.87 Δ*S*_S_ + 85.464	370.87

The general equations Δ*H*_S_ = *f*(Δ*S*_S_) of graphenes deduced from Table 4 can be written as follows:

18 Δ*H*_S_ = *T*_S_Δ*S*_S_ + *Q*

where the slope *T*_S_ represents an isokinetic surface temperature at which all processes in the series of organic solvents proceed with the same work of adhesion on graphene material, here given by the constant parameter *Q*. Eqn (13) then corresponds to the surface enthalpy–surface entropy compensation for the various graphene surfaces.

To compare between the work of adhesion *W*_a_(*T*) of a solvent on graphene materials, the variations of *W*_a_(*T*) of each solvent on the different graphenes as a function of temperature were plotted in Fig. S2. It was showed that the adhesion of all solvents on graphenes satisfied the same classification. The increasing order of the work of adhesion on graphenes is given as follows:Graphene oxide < Reduced graphene oxide < Graphene

The observed trend in the work of adhesion of various solvents on graphene-based materials reflects the fundamental changes in surface chemistry and electronic structure across these materials, directly influencing intermolecular interactions with adsorbing solvent molecules. Indeed, graphene oxide (GO) contains a high density of oxygenated functional groups (hydroxyl, epoxy, carbonyl, carboxyl), which increase polarity and surface heterogeneity but decrease π-electron density and planar conjugation. These polar sites may disrupt van der Waals (particularly London dispersion) interactions, which are dominant in nonpolar solvent adhesion. Whereas reduced graphene oxide (rGO) is partially restored to a graphitic structure with fewer oxygen groups and a partially recovered π-conjugated system, increasing π–π interactions and improving London dispersion forces, especially with nonpolar or slightly polar solvents. While graphene (G) has a fully conjugated, delocalized π-electron system, enabling maximum van der Waals interactions, especially London dispersion with alkane solvents, and π–π or dipole–π interactions with polar solvents such as THF, acetone, and acetonitrile. These results can be interpreted in term of the work of adhesion. Knowing that the work of adhesion *W*_a_(*T*) given by eqn (6) between a solvent and a solid surface is generally a function of the interfacial free energy. Graphene has the highest surface energy due to strong cohesive π–π forces, while GO has the lowest because of disrupted π-systems and hydrophilic heterogeneity, reducing solvent–surface affinity. Thus, higher surface energy in graphene enhances interaction strength and thus adhesion. This is consistent with the classification of solvents. Despite the chemical diversity of the solvents (aliphatic, chlorinated, etheric, carbonyl-containing, nitriles), the same adhesion trend across all of them suggests that the surface nature of the graphene material dominates over solvent-specific properties and the main interaction mechanism is non-specific, *i.e.*, driven largely by van der Waals (especially London dispersion) and π–π stacking, rather than hydrogen bonding or strong dipole–dipole interactions. This explains why nonpolar solvents (*e.g.*, *n*-alkanes) and polar aprotic solvents (*e.g.*, acetone, acetonitrile) follow the same trend. Therefore, the increasing adhesion from GO to graphene is governed by increasing surface energy, enhanced π–electron delocalization, and stronger London dispersion and π–π interactions, which collectively improve the solvent–surface affinity regardless of the solvent's polarity.

## Conclusions

This study comprehensively investigated the temperature-dependent surface adhesion behaviors of graphene (G), reduced graphene oxide (rGO), and graphene oxide (GO) with a variety of polar and non-polar solvents. Through inverse gas chromatography (IGC) and thermodynamic decomposition of work of adhesion into dispersive and polar components, a consistent trend of work of Adhesion was established:Graphene > rGO > GO

• The high dispersive component of pristine graphene enables stronger interaction with solvents dominated by van der Waals forces.

• rGO, being partially restored in its conjugated π-system, shows moderate affinity due to a balance between polar and dispersive interactions.

• GO, rich in oxygen-containing groups, is predominantly polar but exhibits limited dispersive interaction.

The results also highlight that solvent-specific interaction rankings vary depending on the surface polarity and π-electron density of the carbon material. This work underlines the necessity of tailoring solvent-material pairs for applications such as film transfer, nanofluidics, and composite fabrication.

## Conflicts of interest

There are no conflicts to declare.

## Supplementary Material

RA-015-D5RA03892H-s001

## Data Availability

All data presented in this study are available in the article and SI materials. This supplementary information gives the values of polar surface energy, dispersive work of adhesion, and polar work of adhesion of various solvents adsorbed on graphene G, graphene oxide GO, and reduced graphene oxide rGO as a function of temperature. It includes the comparison between graphenes giving the variations of the total work of adhesion of solvents on graphenes *versus* the temperature. *n*-Hexane, *n*-heptane, *n*-octane, *n*-nonane, dichloromethane, diethyl ether, THF, ethyl acetate, acetone, and acetonitrile. See DOI: https://doi.org/10.1039/d5ra03892h.
