# Supplementary material for: Temperature-controlled surface adhesion in graphene materials: experimental trends, surfaces, and interfaces physical chemistry
Source: RSC Adv. 2025 Aug 6;15(34):27941–50. doi: 10.1039/d5ra03892h (PMC12327492; doi:10.1039/d5ra03892h)
Supplement: RA-015-D5RA03892H-s001 [file RA-015-D5RA03892H-s001.pdf]

Supplementary Information (SI) for RSC Advances.
This journal is © The Royal Society of Chemistry 2025

Supplementary Materials

Temperature-Controlled Surface Adhesion in Graphene Materials: Experimental Trends, Surfaces, and Interfaces Physical Chemistry

Tayssir Hamieh [1,2,3]*

[1] Faculty of Science and Engineering, Maastricht University, P.O. Box 616, 6200 MD Maastricht, The Netherlands; t.hamieh@maastrichtuniversity.nl
[2] Institut de Science des Matériaux de Mulhouse, Université de Haute-Alsace, CNRS, IS2M UMR 7361, F-68100 Mulhouse, France
[3] Laboratory of Materials, Catalysis, Environment and Analytical Methods (MCEMA), Faculty of Sciences, Lebanese University, Beirut P.O. Box 6573/14, Lebanon
* Correspondence: t.hamieh@maastrichtuniversity.nl

Table S1. Values of the polar surface energy $\gamma_l^p(T)$ of solvents graphenes as a function of temperature.

**Polar surface energy $\gamma_l^p(T)$ (mJ/m²) of solvents adsorbed on graphene**						
T(K)	Dichloromethane	Diethyl ether	THF	Ethyl acetate	Acetone	Acetonitrile
313.15	3.93	10.86	7.15	8.46	9.46	13.99
323.15	4.12	9.90	6.61	8.36	9.19	14.68
333.15	4.32	8.99	6.07	8.26	8.90	15.40
343.15	4.53	8.12	5.56	8.15	8.62	16.16
353.15	4.75	7.29	5.06	8.03	8.32	16.97
363.15	4.98	6.50	4.58	7.91	8.02	17.82
373.15	5.23	5.75	4.11	7.78	7.71	18.72
**Polar surface energy $\gamma_l^p(T)$ (mJ/m²) of solvents adsorbed on rGO**						
T(K)	Dichloromethane	Diethyl ether	THF	Ethyl acetate	Acetone	Acetonitrile
313.15	3.16	13.37	6.91	10.51	8.37	6.66
323.15	3.34	12.55	6.41	10.30	8.99	7.81
333.15	3.54	11.75	5.92	10.08	9.67	9.10
343.15	3.75	10.96	5.43	9.83	10.41	10.59
353.15	3.98	10.19	4.95	9.58	11.22	12.28
363.15	4.24	9.43	4.47	9.30	12.12	14.20
373.15	4.51	8.69	4.00	9.01	13.12	16.41
**Polar surface energy $\gamma_l^p(T)$ (mJ/m²) of solvents adsorbed on GO**						
T(K)	Dichloromethane	Diethyl ether	THF	Ethyl acetate	Acetone	Acetonitrile
313.15	1.33	23.67	14.19	24.89	23.70	47.06
323.15	1.63	18.63	11.22	21.17	20.87	42.50

333.15	1.93	15.01	9.08	18.46	18.83	39.27
343.15	2.25	12.31	7.48	16.39	17.29	36.88
353.15	2.58	10.23	6.24	14.78	16.13	35.15
363.15	2.92	8.59	5.26	13.48	15.21	33.87
373.15	3.27	7.27	4.46	12.42	14.48	32.89

Table S2. Values of dispersive work of adhesion $W_a^d(T)$ of solvents on graphenes (mJ/m^2) as a function of temperature.

**Dispersive work of adhesion of solvents on graphene (mJ/m²)**							
Temperature T(K)	313.15	323.15	333.15	343.15	353.15	363.15	373.15
n-hexane	134.93	126.33	117.79	109.33	100.93	92.61	84.36
n-heptane	142.43	133.97	125.58	117.26	109.01	100.84	92.74
n-octane	148.34	139.95	131.62	123.36	115.16	107.04	99.00
n-nonane	153.30	144.93	136.61	128.36	120.17	112.04	103.99
CH2Cl2	168.57	158.28	148.06	137.93	127.89	117.94	108.09
Diethyl ether	130.66	122.55	114.50	106.52	98.61	90.77	83.00
THF	165.88	156.32	146.83	137.42	128.09	118.84	109.68
Ethyl acetate	147.91	140.15	132.44	124.78	117.17	109.61	102.11
Acetone	143.16	135.14	127.17	119.27	111.43	103.66	95.95
Acetonitrile	147.67	137.75	127.91	118.14	108.44	98.82	89.27
**Dispersive work of adhesion of solvents on reduced graphene oxide (mJ/m²)**							
T(K)	313.15	323.15	333.15	343.15	353.15	363.15	373.15
n-hexane	99.22	94.87	90.41	85.86	81.18	76.37	71.43
n-heptane	104.74	100.61	96.39	92.09	87.68	83.16	78.53
n-octane	109.08	105.10	101.02	96.88	92.64	88.28	83.82
n-nonane	112.73	108.84	104.85	100.80	96.66	92.40	88.05
CH2Cl2	123.96	118.86	113.64	108.32	102.87	97.26	91.52
Diethyl ether	96.08	92.03	87.88	83.65	79.32	74.85	70.28
THF	121.98	117.39	112.70	107.92	103.03	98.00	92.87
Ethyl acetate	108.77	105.25	101.65	97.99	94.25	90.40	86.46
Acetone	105.28	101.49	97.61	93.67	89.63	85.48	81.25
Acetonitrile	108.59	103.45	98.17	92.78	87.23	81.50	75.59
**Dispersive work of adhesion of solvents on graphene oxide (mJ/m²)**							
T(K)	313.15	323.15	333.15	343.15	353.15	363.15	373.15
n-hexane	87.81	81.73	75.75	69.84	64.04	58.33	52.72
n-heptane	92.69	86.67	80.75	74.91	69.16	63.51	57.96
n-octane	96.54	90.54	84.64	78.81	73.07	67.42	61.87
n-nonane	99.76	93.76	87.85	82.00	76.24	70.57	64.99
CH2Cl2	109.71	102.40	95.21	88.12	81.14	74.29	67.55
Diethyl ether	85.03	79.29	73.63	68.05	62.56	57.17	51.87

THF	107.95	101.13	94.42	87.79	81.27	74.85	68.54
Ethyl acetate	96.26	90.67	85.16	79.72	74.34	69.04	63.81
Acetone	93.17	87.43	81.78	76.20	70.70	65.29	59.97
Acetonitrile	96.10	89.12	82.25	75.47	68.80	62.24	55.79

Table S3. Values of polar work of adhesion $W_a^p(T)$ of solvents on graphenes (mJ/m^2) as a function of temperature.

**Polar work of adhesion of solvents on graphene (mJ/m^2)**							
Temperature T(K)	313.15	323.15	333.15	343.15	353.15	363.15	373.15
CH2Cl2	59.01	58.92	58.84	58.76	58.68	58.60	58.52
Diethyl ether	98.13	91.41	84.94	78.71	72.72	66.93	61.35
THF	79.66	74.65	69.81	65.12	60.58	56.18	51.91
Ethyl acetate	86.60	83.98	81.40	78.84	76.32	73.83	71.36
Acetone	91.58	88.03	84.53	81.08	77.69	74.35	71.06
Acetonitrile	111.39	111.27	111.16	111.05	110.94	110.83	110.73
**Polar work of adhesion of solvents on reduced graphene oxide (mJ/m^2)**							
T(K)	313.15	323.15	333.15	343.15	353.15	363.15	373.15
CH2Cl2	46.40	46.02	45.65	45.27	44.90	44.54	44.17
Diethyl ether	95.47	89.23	83.22	77.43	71.85	66.47	61.28
THF	68.66	63.78	59.06	54.48	50.05	45.76	41.59
Ethyl acetate	84.67	80.85	77.07	73.33	69.64	66.00	62.40
Acetone	75.56	75.53	75.48	75.44	75.40	75.35	75.31
Acetonitrile	67.41	70.37	73.25	76.08	78.86	81.57	84.23
**Polar work of adhesion of solvents on graphene oxide (mJ/m^2)**							
T(K)	313.15	323.15	333.15	343.15	353.15	363.15	373.15
CH2Cl2	10.53	12.47	14.39	16.32	18.21	20.06	21.90
Diethyl ether	44.36	42.21	40.14	38.18	36.26	34.41	32.62
THF	34.34	32.75	31.22	29.76	28.32	26.93	25.57
Ethyl acetate	45.49	44.99	44.51	44.05	43.58	43.11	42.64
Acetone	44.39	44.67	44.96	45.25	45.52	45.79	46.05
Acetonitrile	62.55	63.75	64.93	66.08	67.21	68.32	69.40

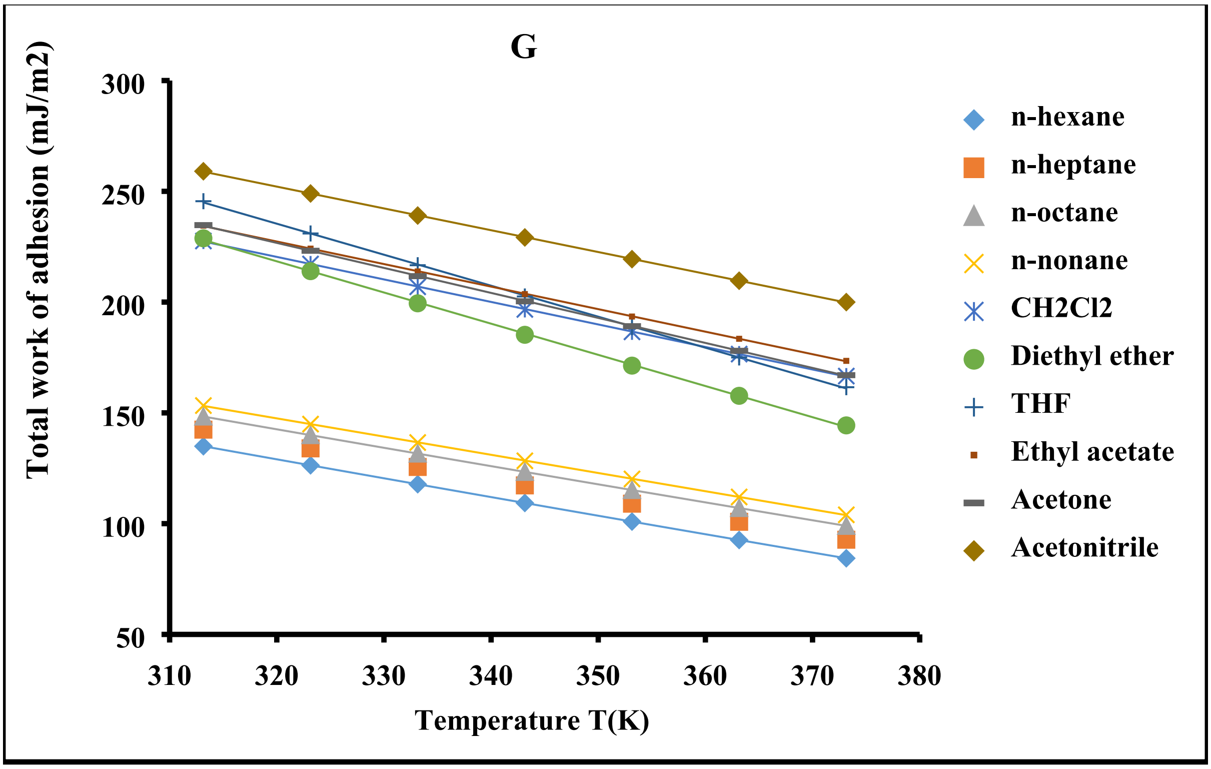
G
Total work of adhesion (mJ/m2)
300
250
200
150
100
50
310
320
330
340
350
360
370
380
Temperature T(K)
n-hexane
n-heptane
n-octane
n-nonane
CH2Cl2
Diethyl ether
THF
Ethyl acetate
Acetone
Acetonitrile

(a)

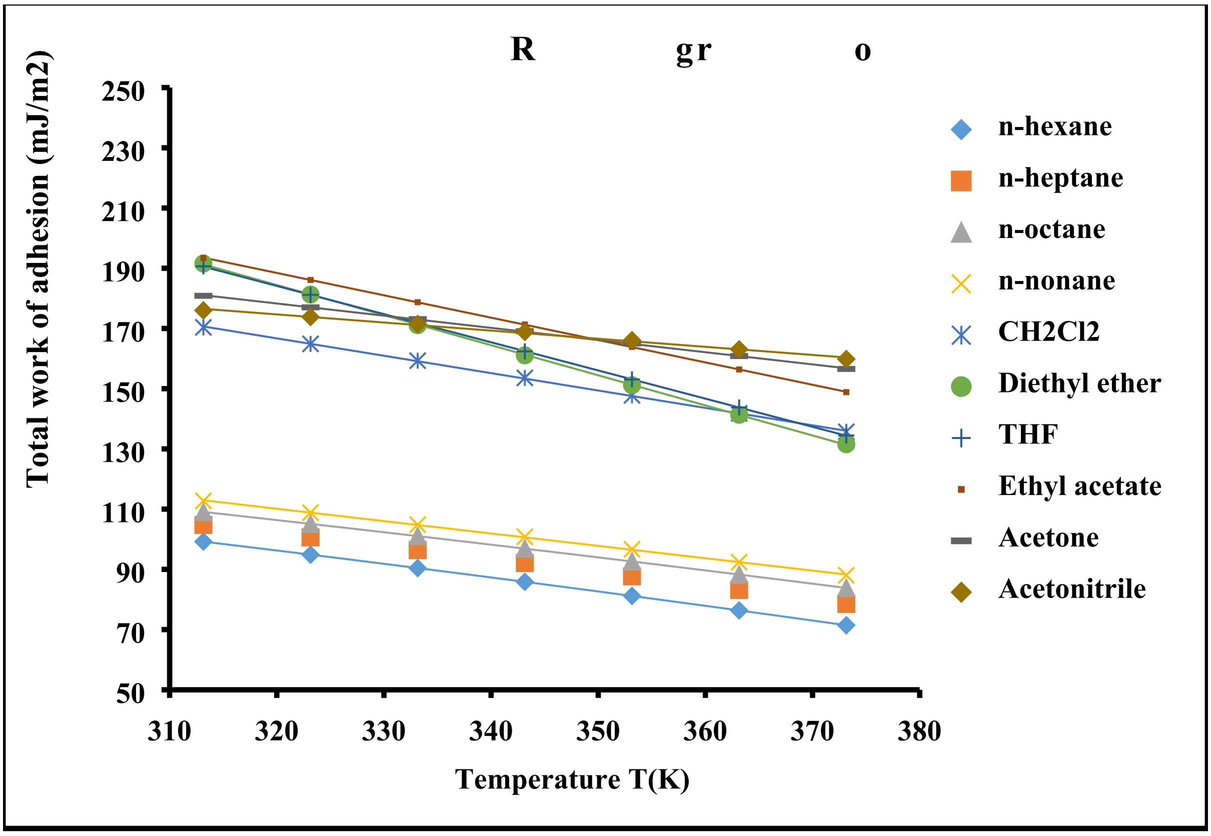
R gr o
Total work of adhesion (mJ/m2)
250
230
210
190
170
150
130
110
90
70
50
310
320
330
340
350
360
370
380
Temperature T(K)
n-hexane
n-heptane
n-octane
n-nonane
CH2Cl2
Diethyl ether
THF
Ethyl acetate
Acetone
Acetonitrile

(b)

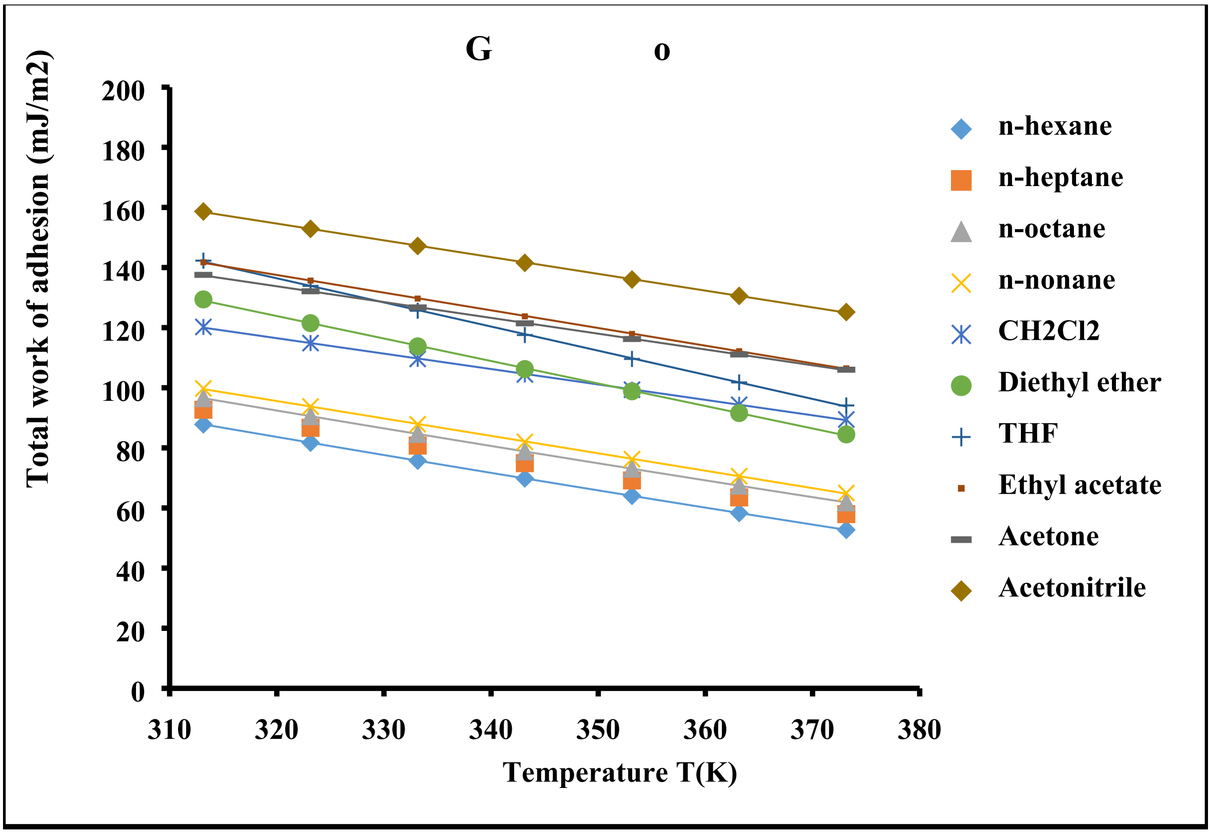

(c)

Figure S1. Variations of the work of adhesion $W_a(T)$ (mJ/m^2) of solvents on graphenes versus the temperature. Graphene (a), reduced graphene oxide (b), and graphene oxide (c).

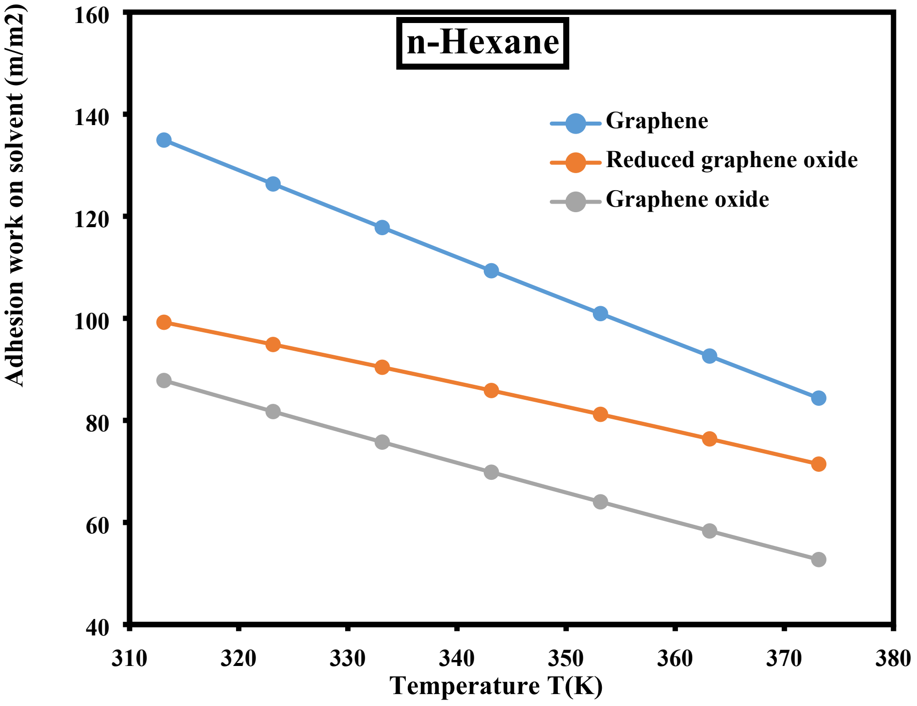

(a)

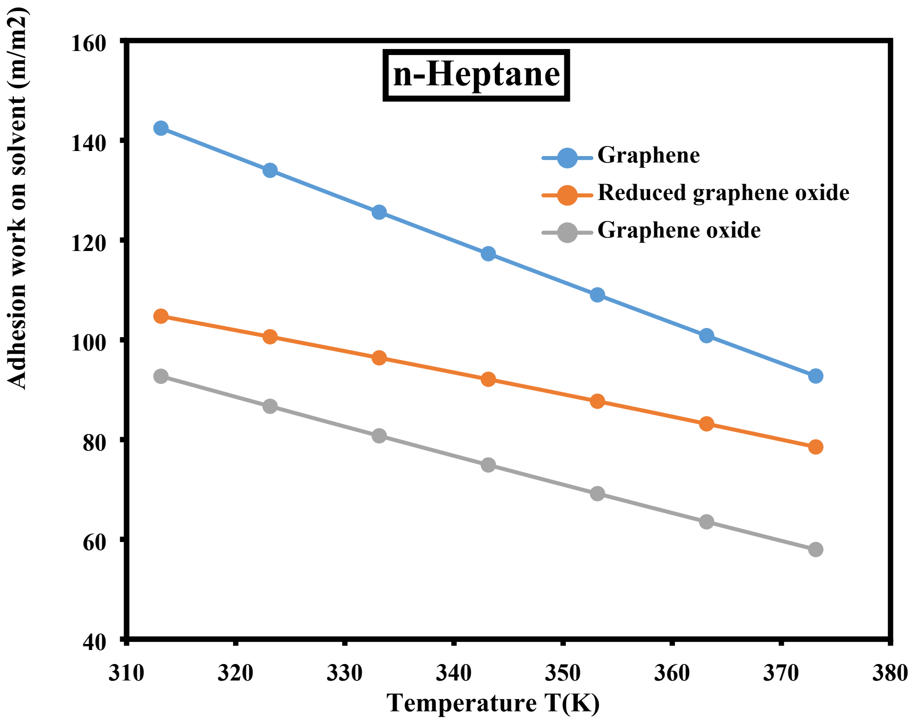
n-Heptane
Graphene
Reduced graphene oxide
Graphene oxide
Adhesion work on solvent (m/m2)
Temperature T(K)
160
140
120
100
80
60
40
310
320
330
340
350
360
370
380

(b)

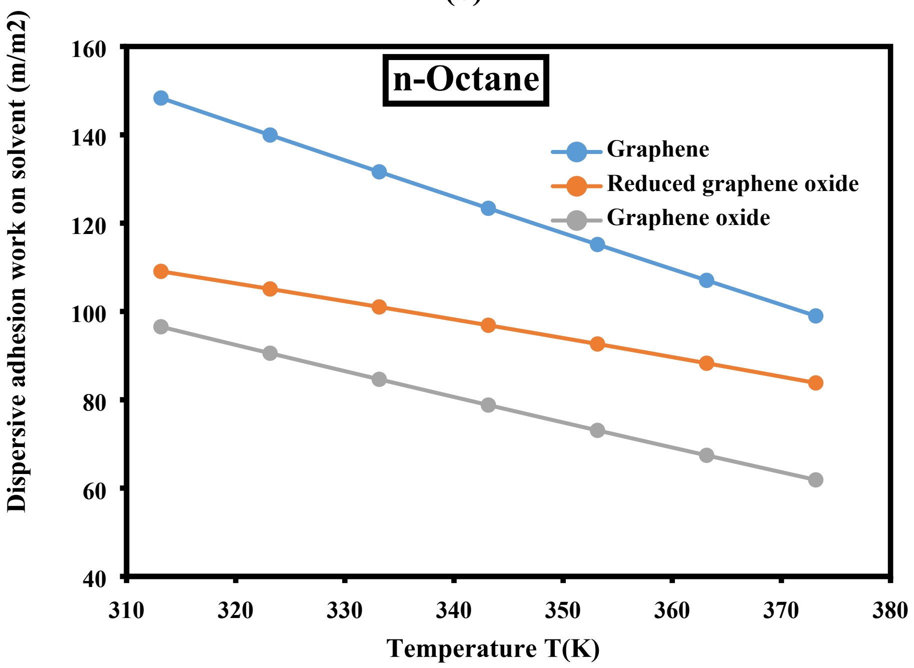
n-Octane
Graphene
Reduced graphene oxide
Graphene oxide
Dispersive adhesion work on solvent (m/m2)
Temperature T(K)
160
140
120
100
80
60
40
310
320
330
340
350
360
370
380

(c)

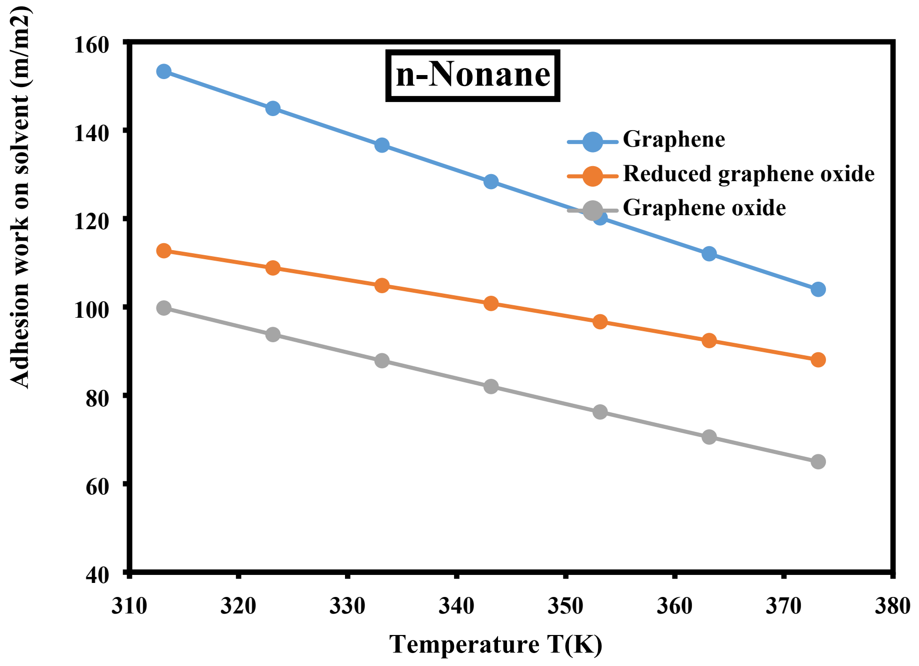
n-Nonane
Graphene
Reduced graphene oxide
Graphene oxide
Adhesion work on solvent (m/m2)
160
140
120
100
80
60
40
310
320
330
340
350
360
370
380
Temperature T(K)

(d)

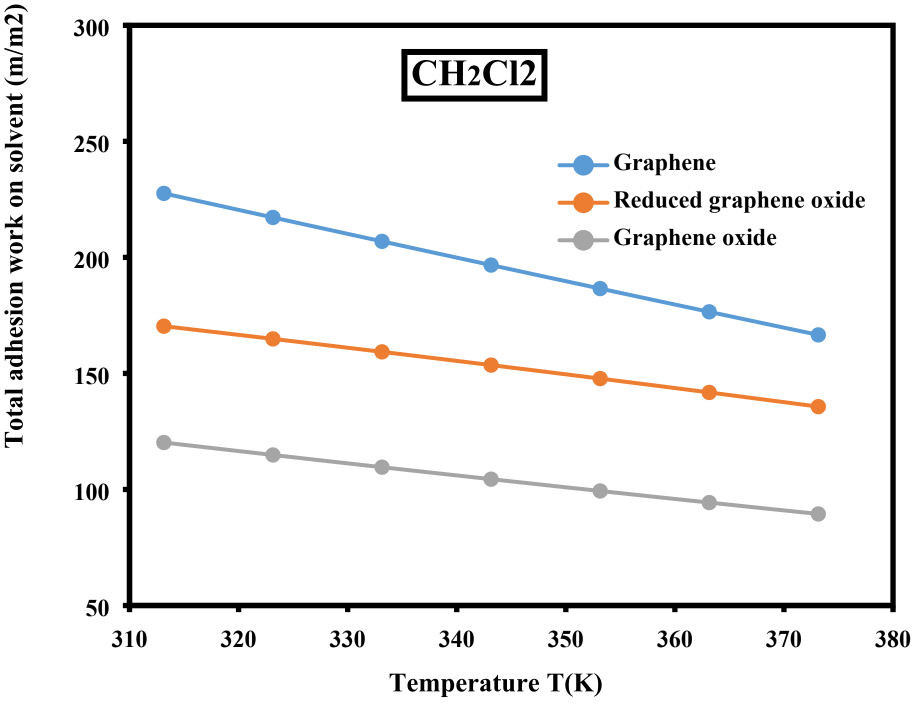
CH2Cl2
Graphene
Reduced graphene oxide
Graphene oxide
Total adhesion work on solvent (m/m2)
300
250
200
150
100
50
310
320
330
340
350
360
370
380
Temperature T(K)

(e)

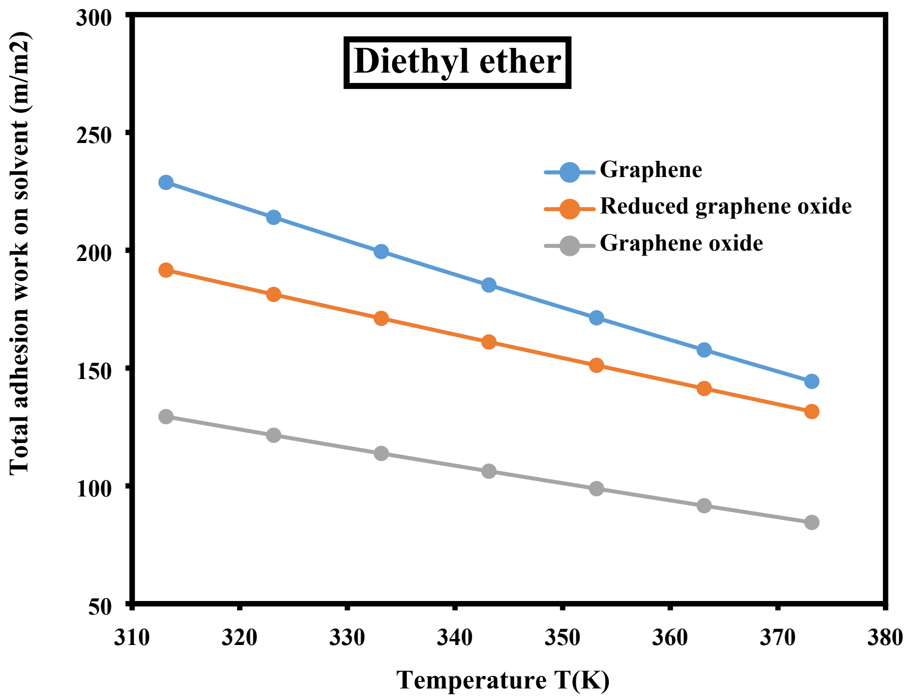
Diethyl ether
Graphene
Reduced graphene oxide
Graphene oxide
Total adhesion work on solvent (m/m2)
300
250
200
150
100
50
310
320
330
340
350
360
370
380
Temperature T(K)

(f)

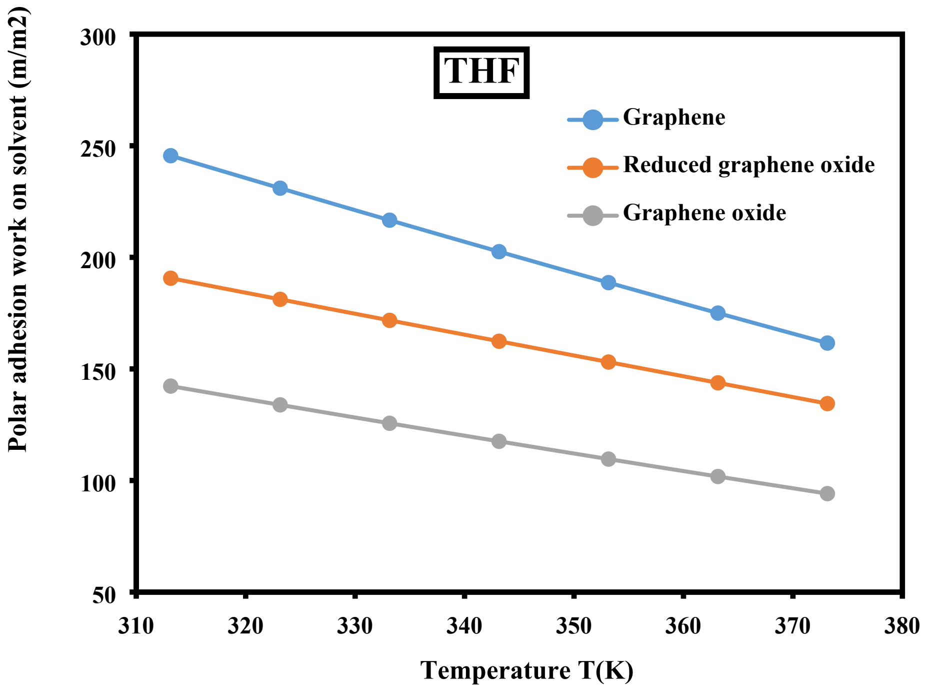
THF
Graphene
Reduced graphene oxide
Graphene oxide
Polar adhesion work on solvent (m/m2)
300
250
200
150
100
50
310
320
330
340
350
360
370
380
Temperature T(K)

(g)

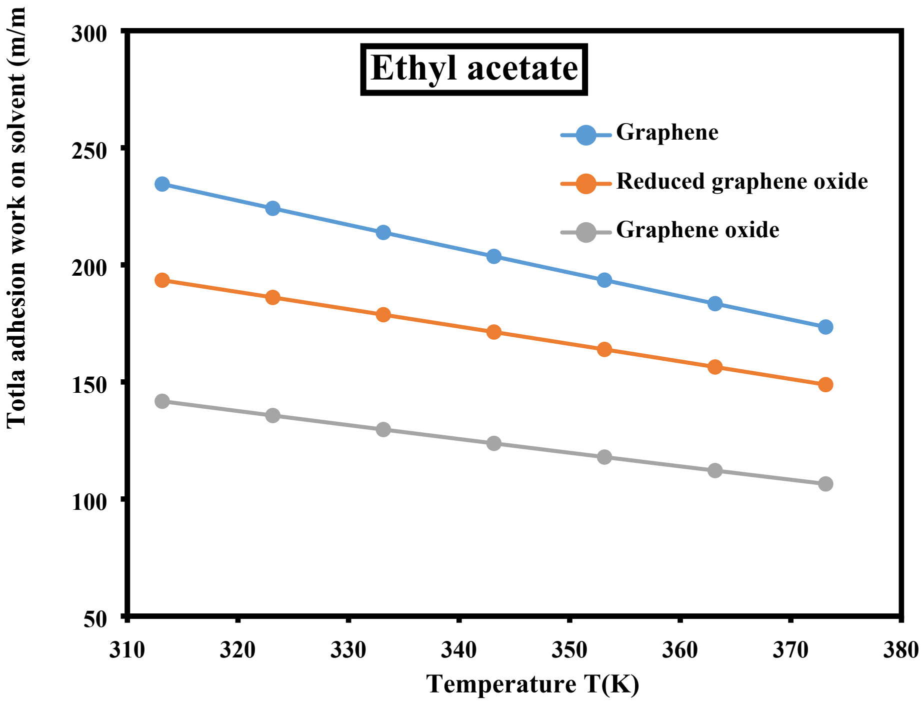
Ethyl acetate
Graphene
Reduced graphene oxide
Graphene oxide
Totla adhesion work on solvent (m/m
300
250
200
150
100
50
310
320
330
340
350
360
370
380
Temperature T(K)

(h)

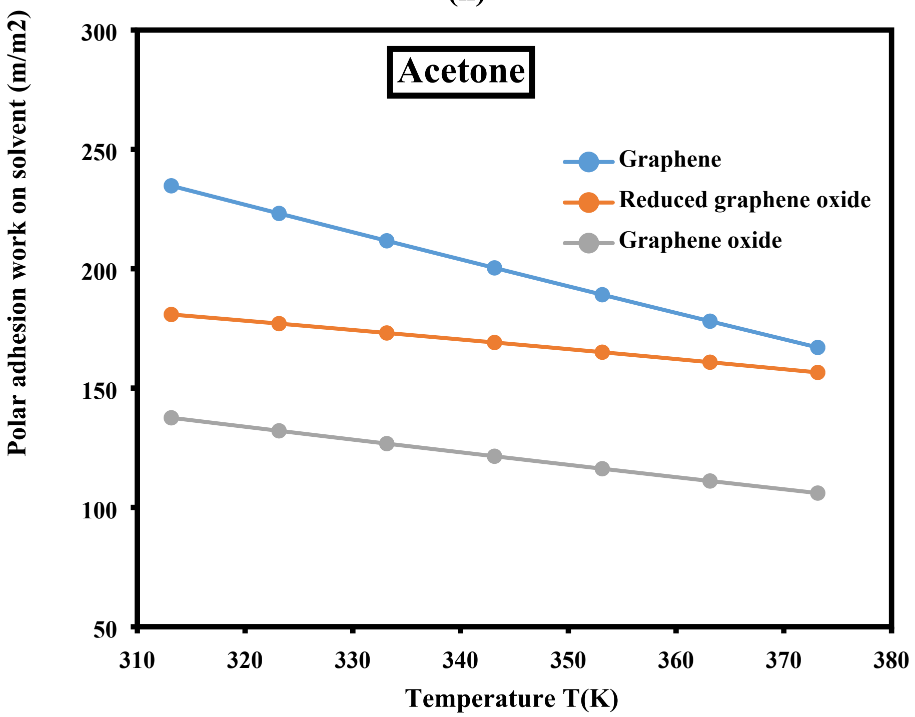
Acetone
Graphene
Reduced graphene oxide
Graphene oxide
Polar adhesion work on solvent (m/m2)
300
250
200
150
100
50
310
320
330
340
350
360
370
380
Temperature T(K)

(i)

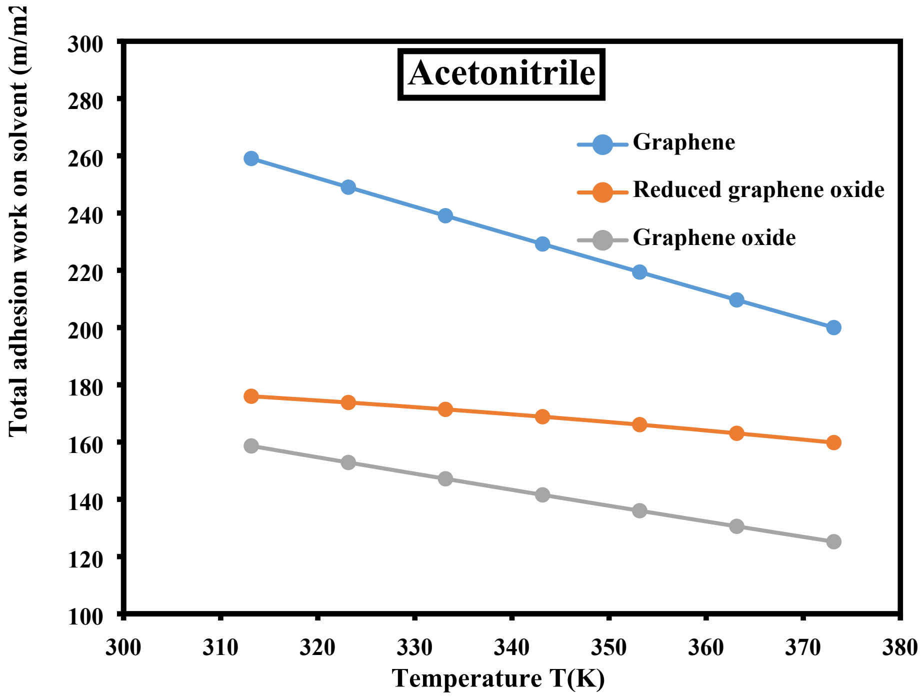

(j)

Figure S2. Variations of the work of adhesion $W_a(T)$ (mJ/m^2) of solvents on graphenes versus the temperature. n-hexane (a), n-heptane (b), n-octane (c), n-nonane ((d), dichloromethane (e), diethyl ether (f), THF (g), Ethyl acetate (h), acetone (i), and acetonitrile (j).
